# SCREENING AND OUTCOMES OF CO-OCCURRING TRAUMATIC BRAIN INJURY AMONG PEOPLE WITH SPINAL CORD INJURY: A SCOPING REVIEW

**DOI:** 10.2340/jrm.v57.41897

**Published:** 2025-01-03

**Authors:** Deborah L. SNELL, Phoebe WYNANDS, Jennifer A. DUNN, Joanne NUNNERLEY, Alice THEADOM

**Affiliations:** 1 Department of Orthopedic Surgery and Musculoskeletal Medicine, University of Otago, Christchurch; 2 Department of Psychology and Neuroscience and the TBI Network, Auckland University of Technology, Auckland, New Zealand

**Keywords:** traumatic brain injury, traumatic spinal cord injury, dual diagnosis, screening, rehabilitation outcomes

## Abstract

**Objective:**

To map existing knowledge on screening and rehabilitation outcomes for co-occurring traumatic brain injury among people with traumatic spinal cord injury (SCI).

**Methods:**

Articles focusing on screening and rehabilitation outcomes in participants sustaining co-occurring traumatic brain injury and traumatic spinal cord injury (all ages) were identified in Ovid, Scopus, Web of Science, CINAHL, and ProQuest Dissertations and Theses electronic databases. There were no limitations on study design, date, or geographical location. Articles were excluded if they were not available in English. Data were extracted into the Rayyan online collaboration platform and summarized descriptively.

**Results:**

Twenty-five studies were included, with a mix of retrospective, case-control, and prospective cohort designs. Screening under-estimated traumatic brain injury incidence when approaches relied on inconsistently collected traumatic brain injury indicators, especially for mild traumatic brain injury. Rehabilitation outcomes included length of stay, functional outcomes, cognitive functioning, complication rates, and employment. Although mixed, outcomes among persons with moderate to severe co-occurring traumatic brain injury especially, appeared poorer than those with spinal cord injury alone.

**Conclusions:**

Multivariable approaches to traumatic brain injury ascertainment and greater consistency in documenting acute traumatic brain injury indicators may improve reliability of capturing traumatic brain injury and traumatic brain injury severity among persons with traumatic spinal cord injury. Impacts of co-occurring traumatic brain injury appear greater relative to SCI alone but few studies analysed outcomes based on traumatic brain injury severity.

## LAY ABSTRACT

Traumatic brain injury, when this occurs with traumatic spinal cord injury, is often referred to as a dual diagnosis. Dual diagnosis, while often missed, is common and rates vary associated with difficulties consistently identifying the traumatic brain injury, especially when the focus is on the traumatic spinal cord injury. In this study we aimed to map what studies say first about how best to identify a co-occurring traumatic brain injury and second about impacts of the traumatic brain injury on traumatic spinal cord injury outcomes. Findings showed greater consistency is needed in the tools used to identify and document the traumatic brain injury. Impacts of co-occurring traumatic brain injury appear greater relative to spinal cord injury alone but only a few studies analysed outcomes based on traumatic brain injury severity. The co-occurring traumatic brain injury has been associated with increased length and cost of traumatic spinal cord injury rehabilitation, smaller gains from admission to discharge, and added difficulties adjusting and returning to activities such as work.

Co-occurring traumatic brain injury (TBI) is common among people who sustain traumatic spinal cord injury (tSCI) and is often referred to as a dual diagnosis (1). TBI and tSCI share similar mechanisms of injury including falls, motor vehicle accidents, and sport-related injuries (2, 3). Cervical tSCIs have been associated with an eventual dual diagnosis of tSCI and TBI (4, 5). Estimated rates of co-occurring TBI among people with tSCI vary, and range from 12 to 74% based on the diagnostic criteria utilized and characteristics of samples across studies (1, 6, 7). Mild TBI, also known as concussion, is common, with some studies finding mild TBI in 64–82% of persons with dual diagnoses (4, 5, 8).

The diagnosis of co-occurring TBI, especially mild TBI, may be missed in the acute care and rehabilitation setting (9). Initial assessment for TBI can include assessment of 1 or more of the following modalities: loss of consciousness (LOC), Glasgow Coma Scale (GCS) score, duration of post-traumatic amnesia (PTA), neuropsychological testing, or computed tomography (CT). Variability in the application of these methods may contribute to difficulties accurately identifying dual diagnosis (4). Further, accurate evaluation of subtle, absent, or masked neurological signs and symptoms may be challenging in a context of more pressing life-threatening injuries (10). In addition, and especially for mild TBI, imaging modalities tend to lack sensitivity and there can be failure to accurately record GCS/LOC/PTA duration in clinical practice. Often there are overlaps of symptoms of mild TBI and emotional responses and treatment effects associated with the tSCI (9, 11). Adding further complexity, cognitive impairments after tSCI may not be specific to TBI and can be attributed to a range of factors other than TBI such as pain, fatigue, medications, impacts of the spinal cord injury, and other systemic features (12, 13). Consequently, some studies have found that TBI can be missed in up to half of persons with tSCI (11).

Missed diagnoses of co-occurring TBI among people with tSCI may have considerable downstream impacts. These impacts have been consistently shown for severe TBI, including increased length and cost of rehabilitation, additional complexities for psychosocial functioning and adjustment and engagement in rehabilitation (14). Some symptoms that are highly prevalent in the early months after even mild TBI, such as persistent cognitive fatigue, dizziness, or emotional dysregulation (15–17), may interfere with participation in rehabilitation. However, mild TBI impacts may be less obvious in people with tSCI, who may be on medications that may also mimic/cause these symptoms or who are not yet engaging in activities where these difficulties may be noticeable. Therefore, diagnosis of co-occurring TBI of any severity in a person with tSCI is important for planning rehabilitation interventions to maximize functional outcomes and prevent further complications (9).

This scoping review aimed to map existing knowledge on screening for co-occurring TBI among persons with tSCI. Given diversity in rates of co-occurring TBI among persons with tSCI across studies, a map of existing screening approaches may shed light on new ways forward to improve consistency of capturing TBI. We then aimed to summarize knowledge on impacts of dual diagnosis on rehabilitation outcomes. While impacts of severe TBI may be obvious, drawing together information on impacts of co-occurring mild and moderate TBI as well may highlight knowledge gaps in the rehabilitation of persons with dual diagnosis.

## METHODS

### Review questions

1. What is the extent and type of evidence relating to screening for co-occurring TBI among persons with tSCI? For example, what is the optimal timing and what are the most reliable approaches to screening for co-occurring TBI?
2. How does co-occurring TBI impact on tSCI rehabilitation outcomes and do outcomes differ by TBI severity?

### Design

The scoping review was conducted in accordance with the methodological framework for scoping reviews first proposed by Arksey and O’Malley (18), subsequently adapted by Levac et al. (19) and further advanced by a working group from the Joanna Briggs Institute (20).

### Eligibility criteria

*Participants.* We included studies focusing on participants sustaining co-occurring TBI and tSCI (including people of all ages).

*Concept.* The concept was to understand capture of co-occurring TBI among persons with tSCI, including approaches to screening, identification, and diagnosis. We were particularly interested in differentiating between symptoms that could be attributable to TBI and tSCI and optimal timing for screening. Rehabilitation outcomes included length of stay in rehabilitation, functional gains during rehabilitation, and longer-term outcomes post-discharge from rehabilitation.

*Context.* Research conducted in any geographical location was considered. We were interested in acute and post-acute rehabilitation settings. There were no limitations placed on date to enable capture of all relevant evidence. Only studies published in English were considered, due to translation constraints.

*Types of sources.* All study designs (e.g., trials, observational studies, and qualitative designs and theses) were considered. Published conference abstracts were flagged for subsequent publications but were not included if no full study was available. Editorials, commentaries, and literature reviews were excluded.

### Search strategy

**Fig. 1:**
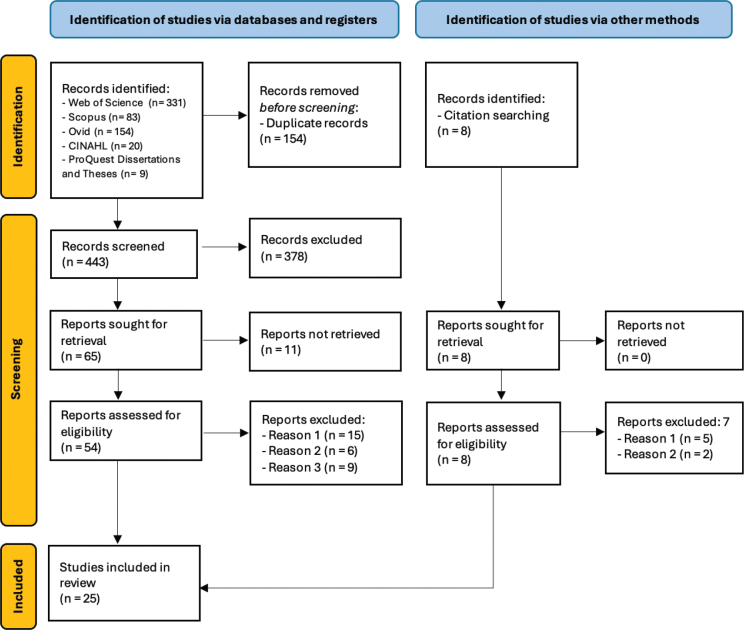
**Preferred Reporting Items for Systematic Reviews and Meta-analyses extension for scoping review (PRISMA-ScR) flow diagram** (Reason 1: background article; Reason 2: wrong population; Reason 3: wrong outcome).

An initial limited search of Web of Science and CINAHL databases was undertaken to identify text words and index terms to develop a full search strategy with the assistance of a subject librarian. The search strategy, including all identified keywords and index terms, was adapted for each database and/or information source. The full search was conducted through the following databases: Ovid, Scopus, Web of Science, CINAHL, and ProQuest Dissertations and Theses. The searches of Ovid, Scopus, Web of Science and ProQuest Dissertations and Theses included the following terms: ((“traumatic brain injur*” OR “brain injur*” OR “concussion” OR “head injur*”) AND (“spinal cord injur*” OR “spinal injur*”) AND (“concurrent” OR “co-occurring” OR “comorbid” OR “dual”) AND ((“diagnos*” OR “screen*” OR “identif*”) OR (“rehabilitation outcome”))). These terms were searched within all fields as keywords, titles and abstracts. This generated 331 results in Web of Science, 83 results in Scopus, and 1,130 results in Ovid; the search within Ovid was then refined to include (“brain injur*” OR “concussion”) AND (“spinal cord injur*”) in titles, yielding 154 results. The ProQuest Dissertations and Theses database was searched to identify any unpublished theses (i.e., Master’s or PhD level) and published conference abstracts to be considered for inclusion (9 results). Citations of all included sources of evidence were then screened for additional studies and the “cited by” search tools were used where available. The terms quadriplegia, tetraplegia, and paraplegia were also checked against TBI search terms to ensure no older literature was missed and one further study was identified for inclusion (Fig. 1).

### Study selection, data extraction, and analyses

Identified citations were collated and uploaded to the Rayyan online collaboration platform (21), and duplicates were detected and removed. At this stage, 2 members of the research team (PW, DS) screened the titles and abstracts independently. Full-text versions of remaining records were then retrieved and reviewed in detail for eligibility against inclusion criteria independently by the same research team members (PW, DS).

Data extracted from included studies were details concerning the study context, participants, aims, methods, outcomes, and key findings relevant to the review questions. These data were summarized into tables to convey the key ideas. In addition, a descriptive summary of included studies provided a map of key findings.

## RESULTS

Identified citations (603) were collated and uploaded to the Rayyan platform, and 154 duplicates were detected and removed. Full texts of 65 records were retrieved. Ten studies were then excluded as only conference abstracts were available. Full texts of the remaining 55 studies were reviewed in detail for eligibility against inclusion criteria. This left 25 studies for inclusion in the review, of which 3 studies addressed both review questions (6, 22, 23). This resulted in 11 studies included for question 1 and 17 studies included for question 2 (Fig. 1).

### Description of studies

Of the 25 included studies (Tables I–III), most used either a cohort (*n* = 15) or cross-sectional (*n* = 8) study design. Two were case-control studies, 9 were either retrospective or prospective, while in 4 studies data were collected both retrospectively and prospectively. There were no qualitative studies. Most studies were conducted in the United States (*n* = 13), followed by Canada (*n* = 5), and then Australia (*n* = 2). There was 1 study each from Brazil, United Kingdom, New Zealand, Finland, and South Korea. Studies that met inclusion criteria were published between 1987 and 2023.

**Table I:** Extent and type of evidence relating to screening for co-occurring TBI among people with tSCI (*n* = 9).

Author/year/country/citation	Aims/purpose	Setting/sample size/population	Injury mechanism and TBI severity	Main methods and study design	Results	Key findings
Bombardier et al. 2016 United States (6)	Diagnostic accuracy of a self-report measure of TBI among people with tSCI	Inpatient rehabilitation n = 105 adults aged 16+ 73% male Mean age 45.9	Vehicular collisions: 40% Falls/flying objects: 44% TBI severity: No TBI: 67% Mild: 91% Complicated mild: 3% Moderate: 6% Severe: 0%	Cross-sectional study Validity of self-report screening measure (TBI-4) compared with medical records review	Estimated incidence of TBI was 33% by chart review and 60% based on TBI-4 TBI-4 overestimated the incidence of TBI by 80%	Poor correspondence between approaches may reflect failure to document TBI indicators or overestimation of self-reported TBI symptoms Participants were confused about whether their symptoms were related to TBI or other factors TBI-4 needs modification to before it could be recommended for clinical use
Budisin et al., 2016 Canada (24)	Compare dual-diagnosis frequency with or without diagnostically ambiguous cases	Tertiary inpatient rehabilitation n = 91 adults aged 18 to 55 73.6% male Median age 37.0	Transport: 42% Fall: 13% Sport: 4% Head vs object: 0.1% Assault: 0% TBI severity: Mean GCS = 13.8	Cross-sectional study Data from medical records (GCS score, PTA, LOC, CT) and MRI Ambiguous cases were missing data confirming TBI	Including ambiguous cases resulted in a difference in prevalence rates of nearly 20% Motor vehicle accidents were 9 times more likely to result in comorbid TBI than other injury mechanisms	Including ambiguous cases, may explain disparate estimates of TBI in tSCI studies Dual diagnosis not less common in thoracic than cervical spine injuries Motor vehicle collisions and falls more likely to result in comorbid TBI
Creasey et al., 2015 United States (25)	Evaluation of people with SCI and TBI attending a Veterans SCI Unit	Veterans Hospital SCI Unit n = 409 adults 97.8% male Mean age 60.0	Transport: 59% Fall: 24% Sport: 12% Assault: 2% Other: 3% TBI severity not reported	Single-centre retrospective study analysing electronic medical records	24% of tSCIs had co-occurring TBI 55% tSCI + TBI had cognitive impairment	Identification of co-occurring TBI inconsistent Improved screening and documentation could improve management
Davidoff et al., 1987 United States (23)	Assessment of TBI among people with tSCI in a rehabilitation setting	Acute and post-acute rehabilitation n = 101 aged 17+	Transport: 38% Fall: 23% Sport: 20% Gunshot/assault: 19% Severity of TBI not reported	Retrospective medical records study seeking presence of LOC and PTA	LOC was assessed in 87% of patients in acute and 67% in post-acute rehabilitation setting; PTA was assessed in 22% of patients in acute and 14% post-acute rehabilitation	Assessment of LOC was performed more consistently than assessment of PTA
Macciocchi et al., 2008 United States (8)	Incidence and severity of co-occurring TBI and tSCI To describe a TBI screening process	Inpatient rehabilitation n = 198 tSCI, ages 16–59 79% male Mean age 28.7	Transport: 63% Fall/flying object: 10% Sport: 13% Violence: 15% TBI severity: Mild: 34% Complicated mild: 10% Moderate: 6% Severe: 10%	Prospective cohort study Data collected on tSCI and TBI from medical records TBI markers included PTA, GCS, LOC, and neuroimaging findings	60% tSCI + TBI LOC (54%) and PTA (58%) GCS scores were unknown/not recorded in 42% of cases Normal CT scan or no CT scan reported (82%) PTA duration most discriminating variable in establishing TBI	A systemic algorithm prioritized PTA before GCS and neuroimaging outcomes Incomplete acute care documentation of TBI remains a considerable issue A systematic algorithm for reviewing acute care medical records may improve estimates of TBI severity
Sharma et al., 2014 Canada (11)	Frequency of missed acute care TBI and risk factors for missed TBI diagnosis	Tertiary inpatient rehabilitation n = 92 adults aged 18 and 55 years with tSCI 74% male Mean age 35.8	Transport: 37% TBI missed in acute care Non-transport: 79% TBI missed in acute care Severity of TBI not reported	Retrospective data (GCS scores, PTA, LOC, and CT) MRI, neuropsychological assessment at 2 to 6 months post-injury Missed TBI identified from medical record review and patient/family reports	41 patients TBI-positive (acute care CT scan 33%, neurological indices 33%, MRI 24%) Of TBI-positive cases, 585% did not receive an acute care TBI diagnosis TBI missed less frequently in motor vehicle accidents than other mechanisms (42.9% vs 75%)	Acute care diagnoses of TBI were missed in more than half of tSCI cases Injury mechanism was a risk factor for missed diagnosis Multiple assessment indicators aided TBI diagnosis
Sikka et al., 2019 United States (22)	Explore TBI screening among SCI patients across the continuum of care	ED, inpatient rehabilitation n = 49 adults with tSCI 81% male Mean age 39.3	Transport: 37.5% Fall: 34% Water Sport: 6% Gunshot 22% TBI severity: None: 35% Mild: 37% Moderate: 6% Severe: 22%	Retrospective review of ambulance, ED, acute trauma care, inpatient rehabilitation notes (GCS, LOC, imaging, PTA, physician admission notes, discharge summaries, ICD-9 TBI codes)	65% with tSCI met criteria for TBI from any source Diagnosis frequency based on mode: 33% from GCS <15; 47% based on LOC; 20% from imaging; 21% from PTA	This study reinforces the inconsistency of detecting TBI among those with tSCI Variable timing of initial TBI identification due to lack of standardised screening
Snell et al., 2022 New Zealand (5)	Feasibility of a diagnostic algorithm for capturing co-occurring TBI	Inpatient rehabilitation n = 51 adults aged 16+ 72.5% male Mean age 49.0	Transport 39% Fall 25% Sport-related 29% Assault 4% Other 4% TBI severity: None: 45% Mild: 35% Complicated mild 10% Moderate 4% Severe 6%	Clinical records audit of admissions to identify co-occurring TBI (mechanism and context of tSCI, PTA, GCS scores, LOC or altered mental state, neuroimaging reports) TBI diagnosis followed algorithm (Macciocchi et al, 2008)	References to PTA evident in <30% of cases Acute GCS score was avail-able in two-thirds of cases Altered mental state, or LOC not recorded in sufficient detail to ascertain TBI Confident determination of TBI made in 39% of cases, 14% were suspected TBI	Application of algorithm not feasible due to inconsistent documentation of TBI indicators Highlights need for a consistent approach to screening for co-occurring TBI among people with tSCI
Tolonen et al., 2007 Finland (26)	Occurrence and severity of traumatic brain injury in patients with tSCI	Post-acute rehabilitation n = 31 adults age 16+ 968% male Mean age 36	Transport: 46% Fall: 42% Other: 12% TBI severity: Mild: 26% Moderate: 35% Severe: 39%	Cross-sectional study Prospective neurological, neuropsychological, and neuroradiological examinations and retrospective record review	74% met diagnostic criteria for TBI 61% sustained LOC or PTA 68% neuropsychological findings linked to TBI 32% trauma-related MRI abnormalities	TBI more frequent in patients with tSCI than documented on hospital admission TBI diagnosis confounded by intoxication at time of injury, hypoxia, medication, complications of physical injuries

TBI: traumatic brain injury; tSCI: traumatic spinal cord injury; MRI: magnetic resonance imaging; GCS: Glasgow Coma Scale score; PTA: post-traumatic amnesia; CT: computed tomography; LOC: loss of consciousness; ED: emergency department.

**Table II:** Studies using primarily neuroradiology approaches to screening for TBI (*n* = 2)

Author/year/country/citation	Aims/purpose	Setting/sample size/population	Injury mechanism and TBI severity	Main methods and study design	Results	Key findings
Jang et al., 2017 South Korea (28)	Evaluating concomitant TBI among people with tSCI, using DTT.	Inpatient rehabilitation *N* = 14 tSCI/TBI aged 22–65 years 92.9% male Mean age 48.9 *N* = 30 sex matched healthy controls Mean age 50.5	Transport: *n* = 9 Fall: *n* = 5 TBI severity: GCS 13-15: *n* = 13 GCS 12: *n* = 1	Retrospective case-control study. DTI performed 47.5 days after tSCI. The CST, CRT, cingulum, and fornix were reconstructed using DTI, and DTI parameters (FA and fibre volume).	FA and fibre volume in the CST, CRT, cingulum, and fornix of the patient group significantly lower than control group (p < 0.05). Lower FA values suggested damaged or less organised white matter.	Lower FA and fibre volume in all 4 neural tracts in patient group compared with control group suggested injury of these neural tracts. Conventional brain MRIs of the patient group were normal. DTI shows promise for detecting TBI changes.
Wei et al., 2008. Canada (29)	Characterize TBI and tSCI cerebral white matter changes to improve diagnostic accuracy of co-occurring TBI.	Inpatient rehabilitation *N* = 22 patients 68.2% male Mean age 34.3 3 participant groups: SCI/TBI (*n* = 7), SCI no TBI (*n* = 15) and healthy controls (*n* = 12)	TBI cases: Transport: *n* = 4 Fall: *n* = 3 TBI severity: No TBI: *n* = 15 Moderate TBI: *n* = 1 Severe TBI *n* = 1 Unknown: *n* = 5 All TBI had positive MRI	MRI in subacute period with subsequent DTI –derived FA data in white matter tracts compared between groups. Between-subject comparisons of FA were performed using region of interest analysis and tract-based spatial statistics.	Compared with controls and SCI only, SCI/TBI had multiple regions with reduced FA, including the forceps minor, splenium and genu of corpus callosum, and anterior limb of internal capsule. No significant regional FA differences were found between the SCI-only group and healthy controls.	DTI shows promise in detecting TBI-related white matter changes associated with diffuse axonal injury in tSCI. No white matter abnormalities were found in SCI only group. Normative data needed before FA values can be used to diagnose TBI on individual basis.

TBI: traumatic brain injury; tSCI: traumatic spinal cord injury; DTI: diffusion tensor imaging; MRI: magnetic resonance imaging; CT: computed tomography; DTT: diffusion tensor tractography; CST: corticospinal tract; CRT: cortico-reticulospinal tract; FA: fractional anisotropy.

**Table III:** Studies evaluating impacts of co-occurring TBI on tSCI rehabilitation outcomes (*n* = 17)

Author/year/country/citation	Aims/purpose	Setting/sample size/population	Main methods and study design	Results	Key findings
Bombardier et al., 2016 United States (6)	Potential effect of comorbid TBI on acute and post-acute outcomes in SCI	Inpatient rehabilitation *n* = 105 73% men; mean age 45.9 Injury mechanism: – Vehicular collisions: 40% – Falls/flying objects: 44% TBI severity: – Mild: 94% – Moderate: 6%	Cross-sectional study tSCI with TBI versus no TBI compared on length of stay, FIM, discharge disposition, and 1-year outcomes	Non-significant trends for longer LOS (*p* = 0.06) and higher functional status (FIM) at discharge (*p* = 0.09) among those with TBI Similar 1-year group outcomes (divorce/employment rates, life satisfaction, depression, alcohol use, past-year history of UTIs, pressure ulcers)	TBIs were mostly mild with limited impact on acute rehabilitation outcomes Rehabilitation clinicians should still be alert to comorbid TBI and provide neuropsychological assessment, interventions, and accommodations when indicated
Boyle et al., 2014 Australia (44)	Factors supporting employment among people with dual diagnosis	Spinal rehabilitation unit *n* = 30 adults with tSCI + TBI 86.7% male; mean age 42.0 Transport mechanism: 60% TBI severity: – Mild–moderate: 27%	Cross-sectional cohort study Participants were in first 5 years post-rehabilitation discharge	At injury 90% were in paid employment; 47% still in paid employment at time of interview No differences in social, physical, or attitudinal supports/barriers between employed and unemployed groups	Few physical or attitudinal barriers and highly supportive interpersonal relationships with close family and friends described The most common facilitator of employment was personal motivation
Bradbury et al., 2008 Canada (30)	Clinical and economic burden of TBI in people with tSCI	Inpatient rehabilitation *n* = 10 tSCI/TBI matched to *n* = 10 SCI-only controls 70% male; mean age 36.1 Injury mechanism: – Transport: *n* = 4 – Fall: *n* = 4 – Sport: *n* = 1 – Blunt force: *n* = 1 TBI severity not reported	Prospective case-control study Outcomes were LOS, healthcare costs, behavioural and critical incidents, FIM, Personality Assessment Inventory, and neuropsychological assessment findings	Increased rehabilitation costs and nursing care in SCI/TBI group Mean LOS for SCI/TBI group 138 days vs 100 days in the SCI-only group (*p* = 0.08) Neuropsychological performance significantly worse in SCI/TBI group More behavioural incidents in the SCI/TBI group	Poorer psychosocial functioning and more severe neuropsychological impairment in SCI/TBI vs SCI-only groups Daily costs of rehabilitation, costs/FIM change, and level of nursing care was greater for SCI/TBI than SCI-only group
Clark et al., 2023 United States (45)	Clinical outcomes and employment status in veterans with and without a dual diagnosis of SCI/TBI	Veteran Program *n* = 12,985 Iraq/Afghanistan-era veterans 91.3% male; mean age 34.7 Injury mechanism: – Transport-related: 44.9% – Fall: 44.9% – Blast: 78.9% – Bullet: 6.3% TBI severity not reported	Cross-sectional study where veterans were grouped as SCI/TBI (1.1%), TBI only (61.6%), and no TBI or SCI (37.3%) Outcomes included neurobehavioral and psychiatric symptoms, pain, employment status	Significant associations between TBI history, regardless of co-occurring SCI, and all clinical outcomes; with comparable levels of neurobehavioral and psychiatric symptoms, pain All 3 groups showed comparable rates of unemployment	Regardless of SCI status, participants with TBI endorsed poorer clinical outcomes than participants without TBI or SCI Effects appeared likely solely due to the TBI
Davidoff et al., 1987 United States (23)	Frequency of cognitive dysfunction, in a group of SCI patients	Inpatient setting *n* = 30 consecutive adults aged 18 or older Injury mechanism: – Transport: *n* = 12 – Falls: *n* = 6 – Gunshot: *n* = 6 – Sport: *n* = 2 – Other: *n* = 4 TBI severity: *n* = 12 LOC	Prospective cohort study Frequency of cognitive dysfunction at 8 and 12 weeks after SCI 3 groups: new TBI = LOC at time of SCI; old TBI = previous TBI but no LOC or PTA at time of SCI; no TBI	*n* = 12 new TBI; *n* = 6 old TBI; *n* = 12 no TBI 57% of all patients had scores suggestive of cognitive dysfunction SCI+TBI (new or old) more cognitive errors	Cognitive dysfunction prevalent at 8–12 weeks following injury Deficits in attention, concentration, visual problem-solving, abstract reasoning, and ability to adapt may impede learning of new skills and information
de Melo Nato et al., 2014 Brazil (31)	Characteristics of those with SCI and TBI	Tertiary rehabilitation centre *n* = 52 all ages 85% male; mean age 38.6 Injury mechanism: – Transport: 71% – Fall: 11% – Sport: 6% – Other: 12% TBI severity: – Mild TBI: 65% – Moderate TBI: 8% – Severe TBI: 27%	Prospective cross-sectional study Participants characterized on symptoms, level of TBI, associated injuries, complications, treatment, length of hospitalization, and deaths	SCI/TBI had significantly longer (*p* = 0.01) LOS (20±28 days) than those with SCI alone (9±12 days) SCI/TBI had more complications than SCI alone SCI/TBI group 2.48 (*p*<0.01) higher risk of death than SCI alone	Hospitalization time was significantly longer in SCI/TBI patients with higher risk of death and complications
Dowler et al., 1997 United States (40)	Cognitive outcomes in a chronic SCI sample	Veteran Medical Center *n* = 91 at least 1-year post-injury (mean 17 years) *n* = 75 healthy control volunteers Injury mechanism not reported TBI severity not reported	Cross-sectional study evaluating cognitive outcomes Cognitive function was assessed with a battery of tests with minimal manual requirements	Six SCI groups with distinct cognitive profiles identified Groups 1–2 normal cognitive function; most no TBI Group 3 TBI-related deficits in attention and processing speed Group 4 deficits in processing speed only Group 5 TBI-related cognitive flexibility deficits Group 6 memory deficits	Clinically meaningful patterns of cognitive outcomes in SCI individuals with TBI Neuropsychological assessment may help to rule in or out a possible TBI
Garlanger et al., 2018 United States (33)	Functional outcomes between persons with dual diagnosis and SCI only	Inpatient rehabilitation *n* = 256 adults; SCI only (59%); SCI/TBI (41%) Male 73.8%; mean age 46.4 Injury mechanism (SCI/TBI): – Transport: 68.3% – Fall: 22% – Sport: 7% – Other: 3% TBI severity (SCI/TBI): – Mild TBI: 57% – Moderate–severe: 43.3%	Retrospective chart review to identify co-occurring TBI Length of stay, discharge location, and FIM scores were compared between groups	Length of stay did not differ significantly between groups Those with moderate–severe TBI had lower FIM scores on admission (*p*<0.01) and discharge (*p*<0.03) and were less likely to discharge to own home (*p*<0.05)	tSCI patients with moderate and severe TBI made fewer gains during inpatient rehabilitation, had cognitive impairments on discharge, and were less likely to discharge to own home
Gober et al., 2023 United States (32)	Comparison of children with SCI/TBI and those with SCI only on age, gender, race, LOS, and hospital charges	Hospital setting *n* = 1,286 children aged 0–18 years hospitalized with SCI *n* = 787 SCI only *n* = 499 SCI/TBI Injury mechanism not reported TBI severity not reported	Retrospective cross-sectional analysis of hospital discharges from US hospitals participating in the Kids’ Inpatient Database	Prevalence of TBI among children with SCI was 39% Average hospital LOS in the SCI group was 4 days, compared with 6 days in the SCI/TBI group Average total charge in the SCI group was US$98,089 compared with US$124,198 in SCI/TBI group	Paediatric SCI/TBI associated with longer hospital LOS and higher health expenditures compared with SCI only
Gordan et al., 2012 United States (37)	Impact of patient characteristics and speech–language pathology (SLP) interventions on outcomes at discharge and 1-year post-tSCI	Inpatient rehabilitation *n* = 1,032 adults Injury mechanism: – Transport: 65% – Fall: 27% – Violence: 2% – Sport: 6% TBI severity not reported	Longitudinal study documenting SLP treatment Discharge and 1-year post-injury outcomes: cognitive FIM, societal participation (CHART), mood (PHQ-9)	42% identified as having co-occurring TBI More SLP services were given to SCI/TBI group Days from injury to rehabilitation admission, older age associated with lower cognitive FIM scores Patient/treatment characteristics did not predict participation or mood at 1-year post-injury	Persons with SCI/TBI receive more SLP cognitive-communication treatment LOS in rehabilitation facility is significantly and positively associated with discharge cognitive FIM
Macciocchi et al., 2013 United States (39)	Cognitive effects of mild TBI in acute physical trauma population	Inpatient rehabilitation at Modal Systems Centre *n* = 117 adults aged 16–59 Injury mechanism: – Transport: 74% – Fall/flying object: 15% – Sport: 8% – Violence: 4% TBI severity: – Mild TBI: 45%	Prospective cohort study comparing cognitive performance among those with SCI/mild TBI and SCI alone between 26 and 76 days post-injury	Primary outcomes visual, verbal, and working memory, perceptual reasoning, and processing speed Mild TBI group did not show greater impairment Predictors of cognitive function were education, race, history of learning problems, and days from injury to rehabilitation admission	Single mild TBI limited effect on cognitive functioning at 26–76 days after SCI Other factors tend to play a more significant role Lower-than-expected cognitive performance in SCI with and without TBI may indicate SCI alone impacts cognition
Macciocchi et al., 2004 United States (34)	Functional gains and LOS among those with SCI/TBI vs SCI only	Acute and post-acute inpatient rehabilitation *n* = 41 with SCI/TBI N = 41 controls (SCI only) 82.9% SCI/TBI male, 87.8% male SCI only; mean age TBI/SCI 366; SCI only 314 Injury mechanism: not reported TBI severity: – Mild TBI: 51% – Moderate TBI: 27% – Severe TBI: 22%	Retrospective comparison study Groups matched on level of SCI and admission Motor FIM score	On admission, both groups had equivalent Motor FIM scores, but SCI/TBI group less Motor FIM change during rehabilitation SCI/TBI group lower Cognitive FIM scores Those with severe TBI greater Cognitive FIM change LOS and cost of rehabili-tation did not statistically differ between groups	SCI/TBI group made smaller functional gains SCI/TBI group showed lower Cognitive FIM on admission and discharge but the change in Cognitive FIM was equivalent to SCI group No significant differences were observed between groups regarding LOS or rehabilitation cost
Macciocchi et al., 2012 United States (14)	Impact of co-occurring TBI on functional and cognition outcomes during acute SCI rehabilitation	Inpatient rehabilitation at Modal Systems Centre *n* = 189 aged 16 to 59 years 78% male; mean age 28.5 Injury mechanism: – Transport: 63% – Fall/flying object: 10% – Sport: 14% – Violence: 15% TBI severity: – No TBI: 41% – Mild TBI: 21% – Moderate TBI: 6% – Severe TBI: 10%	Prospective, longitudinal cohort study FIM, motor and cognitive assessments 1 to 4 weeks post-admission compared between tetraplegia and paraplegia groups	In tetraplegia group, co-occurring TBI not related to FIM Motor scores or LOS, but moderate and severe TBI negatively affected cognitive function Co-occurring severe TBI and paraplegia had lower admission and discharge FIM Motor scores and longer acute rehabilitation LOS	TBI-related functional outcomes differentiated between paraplegia and tetraplegia Paraplegia/severe TBI worse motor and cognitive outcomes, longer LOS Tetraplegia/TBI impacted memory, attention, and problem-solving
Mirzaeva et al., 2020 Russia (43)	Complications, length of stay, and survival after tSCI	Rehabilitation hospital setting *n* = 311 adults 72% male; mean age 42.0 Injury mechanism: – Transport: 19% – Fall: 50% – Sport: 10% – Violence: 6% – Other/unknown: 16% TBI severity not reported	Retrospective record review for demographic and clinical characteristics, complications, and mortality rate	Co-occurring TBI in 40% of tSCIs Those over 60 had lowest rate of TBI (25%) More complications in tSCI/TBI group (42%) vs those without TBI (29%)	Older patients tended to have less severe injuries and TBI was less common Co-occurring TBI was associated with more complications and higher mortality
Mollayeva et al., 2021 Canada (38)	Cognitive outcomes in TBI with and without comorbid SCI groups	Inpatient rehabilitation TBI/SCI (*n* = 1,195) matched to TBI-only (*n* = 2,390) on sex, age, income, in a ratio of 1:2 65.4% male; mean age 61.8 Injury mechanism (SCI/TBI group): – Transport: 43% – Fall: 39% – Struck: 5% – Other: 13% TBI severity: – Unknown: 33% – Mild TBI: 22% – Moderate TBI: 5% – Severe TBI: 41%	Retrospective matched population-based cohort study comparing TBI/SCI with TBI only Outcomes cognitive FIM on admission and discharge controlling for comorbidity, gains in motor function, and rehabilitation care indicators	TBI/SCI group had lower average FIM cognitive functional gain; 2.98±4.74 compared with 3.10±4.72 (TBI-only group) TBI/SCI group gained less during inpatient rehabilitation than matched TBI-only patients, regardless of functional level on inpatient rehabilitation admission	TBI/SCI group showed lower cognitive domain response to inpatient rehabilitation than those with TBI alone (controlling for comorbidity propensity score, gains in motor function) Findings can help with identifying TBI/SCI patients at risk of worse cognitive outcomes to direct treatment
Nott et al., 2014 Australia (35)	Impact of co-occurring TBI-associated cognitive impairments on community reintegration	Specialist rehabilitation setting SCI/TBI adults (*n* = 30) matched to SCI only (*n* = 30) or TBI only (*n* = 30) 878% male; mean age 41.2 years Injury mechanism (SCI/TBI group): – Transport: n = 18 – Falls and sport: *n* = 10 – Other: *n* = 2 TBI severity: – Mild TBI: PTA (<1 day) *n* = 8; GCS (13–15) *n* = 20 – Moderate TBI: PTA (1–7 days) *n* = 2; GCS (9–12) *n* = 3 – Severe TBI: PTA (>7 days) *n* = 20; GCS (3–8) *n* = 4	Cross-sectional, case-matched study with 3 groups Medical history, level of SCI, GCS, PTA, and FIM scores obtained from records Outcome measures medical status, psychological functioning, FIM, social engagement and relationships, and employment status	LOS greater in SCI/TBI and SCI-only groups On discharge all groups showed FIM total score >100 (moderate level of independent functioning), similar levels of psychosocial functioning, community reintegration, relationship, and employment status The SCI/TBI and SCI-only groups achieved comparable FIM cognitive scores and significantly higher FIM cognitive scores than TBI-only group	At 36 years post-injury, adults with SCI/TBI achieved comparable levels of medical, psychological, and functional recovery, employment, and community participation to SCI and TBI peers Longer LOS may have contributed to the positive findings
Ramamurthy et al., 2017 United Kingdom (36)	Duration of inpatient rehabilitation and outcome in people with TBI and SCI	Inpatient rehabilitation *n* = 269 with SCI (*n* = 27 with co-occurring TBI); mean age 42.4 Injury mechanism (SCI/TBI group): – Transport: 55% – Fall: 37% – Sport: 7% TBI severity: – Mild TBI: 48% – Moderate TBI: 33% – Severe TBI: 19%	Retrospective epidemiological observational study Outcomes: rehabilitation outcomes (FIM), LOS, discharge destination, and functional status at latest follow-up	Mild TBI group gained a mean FIM score of 67.1; the moderate and severe TBI groups 60.1 and 69.2, respectively No significant difference in mean LOS by TBI severity; duration was 138.3, 139.4, and 153.4 days for mild, moderate, and severe TBI, respectively	Duration of inpatient rehabilitation not influenced by severity of TBI No differences in FIM gains from rehabilitation by severity of TBI FIM may be insensitive as a measure on its own
Richards et al., 1988 United States (41)	Impact of cognitive deficits over time, deficits due to TBI should show improved performance (“recovery curve”)	Regional spinal centre *n* = 150 >16 years old; no history of psychiatric illness or TBI Injury mechanism: – Transport: 58% – Other: 42% TBI severity: – LOC: 59% – No LOC: 42%	Longitudinal cohort study Cognitive assessments at 7 weeks after SCI *n* = 67 reassessed 38 weeks after initial assessment	Small improvements noted on memory and general intellectual ability, verbal fluency, and rapid-changing conceptual set tasks Improvement for all SCI subjects by 38 weeks Level of injury, aetiology, LOC not good predictors of changes in neuropsychological test scores	Cognitive improvement from 7 to 38 weeks (recovery curve) consistent with mild to moderate TBI recovery expectations
Sikka et al., 2019 United States (22)	Services provided to individuals with SCI/TBI Identify differences in FIM change based on presence or absence of TBI	ED, inpatient rehabilitation *n* = 49 adults with tSCI (65% SCI/TBI) 81% male; mean age 39.3 Injury mechanism: – Transport: 38% – Fall: 34% – Gunshot: 22% – Water sports: 6% TBI severity: – No TBI: 35% – Mild TBI: 37% – Moderate TBI: 6% – Severe TBI: 22%	Retrospective chart review Outcomes: admission and discharge FIMs, discharge disposition	Motor, cognitive, and total FIM scores higher for tSCI without TBI than with TBI on admission and discharge No significant difference in FIM gain or FIM efficiency between SCI/TBI and no-TBI groups	FIM scores support the differing levels of motor and cognitive functioning among the SCI/TBI patients compared with SCI-only

TBI: traumatic brain injury; tSCI: traumatic spinal cord injury; GCS: Glasgow Coma Scale score; PTA: post-traumatic amnesia; LOC: loss of consciousness; FIM: Functional Independence Measure; LOS: length of stay; UTI urinary tract infection; CHART: Craig Handicap Assessment and Reporting Technique; PHQ-9: Patient Health Questionnaire-9; ED: emergency department; SLP: speech–language pathology.

Of the study populations, in 22 studies participants were recruited following admission to either acute hospital care or a rehabilitation facility with SCI (±TBI). In 3 studies, participants were recruited through the United States Veterans Administration Program, and in 1 study participants were recruited from acute hospital care with a TBI (±SCI). Almost all studies (*n* = 24) included adult participants who ranged in age from 16 to 75 years. Only 1 study included child participants aged between 0 and 18 years.

### Evidence relating to screening for co-occurring TBI among persons with tSCI

*Screening based on clinical records and TBI markers (Table I)*. Nine studies evaluated medical records either retrospectively or prospectively to identify TBI among tSCI admissions specifically looking for TBI indicators such as LOC, GCS scores, and PTA (5, 8, 11, 22–26). Severity of the co-occurring TBI was often not reported clearly, but, if severity information was available, TBIs in the mild and severe range were more common than those in the moderate range (5, 6, 8, 22). The most common injury mechanisms were transport-related causes, especially motor vehicle accidents. Co-occurring TBI was less likely to be missed in acute care if the injury mechanism was transport-related, with 37% missed compared with 79% missed from other injury causes (11). However, falls and sport were also common injury mechanisms in dual-diagnosis samples.

Sikka et al. (22) examined the frequency with which TBI was identified based on different severity markers. In their sample (*n* = 49), 37% were categorized as mild, 6% as moderate, and 22% as severe TBI. The GCS was the sole severity indicator documented for all patients but only 50% of the dual-diagnosis group were categorized as having TBI based on this scale alone. Inconsistent documentation was noted across service lines, with only 72% of those with TBI indicators in the ED setting still having TBI listed on the inpatient rehabilitation admission paperwork.

Three studies collected prospective data on study participants in addition to retrospective records review. Budisin et al. (24) reviewed TBI indicators including structural magnetic resonance imaging (MRI), GCS, PTA, LOC, and CT reports, to classify cases as definite positive TBI, definite negative TBI, and ambiguous for TBI. Ambiguous cases were those with missing or unclear TBI indices. No individual investigation had high sensitivity or specificity for TBI in the mild range and the authors recommended persons with any suspicion of TBI should be flagged for later follow-up. Similarly, Sharma et al. (11) added MRI to records reviews and conducted neuropsychological assessments at 2 and 6 months post-injury to identify possible missed TBI in their SCI cohort. Approximately one-third of TBI cases were identified from acute care CT scan, another one-third from neurological indices, 24% from MRI, and the rest identified by neuropsychological testing. When this study employed multiple assessments to aid TBI diagnosis, it allowed detection of TBI in more than half of SCI cases that were missed in acute care. The authors speculated that co-occurring TBI was more likely to be missed when the injury mechanism was other than a motor vehicle accident and the TBI was in the milder range of severity. A study from Finland also used multiple approaches to capturing co-occurring TBI (neurology exam, neuropsychological assessment, MRI) in combination with acute injury markers such as GCS and PTA, in a sample of 31 persons with tSCI (26). Using this multivariable approach, the authors identified a higher frequency of co-occurring TBI (74%) than other studies, with a mix of mild (26.1%), moderate (34.8%), and severe (39.1%) TBIs.

Macciocchi et al. (8) again noted the problem of inconsistent documentation of TBI indicators. In their sample (*n* = 198), 60% sustained a co-occurring TBI (44% mild or mild complicated, 16% moderate–severe). Motor vehicle accidents and falls were associated with higher rates of co-occurring severe TBI. The authors felt PTA duration was the most discriminating variable in establishing a TBI diagnosis and developed a systematic algorithm that prioritized estimated duration of PTA before tracking variations associated with neuroimaging outcomes and the GCS score. Snell et al. (5) then applied this algorithm; however, this was difficult to apply because of inconsistent documentation of TBI indicators such as PTA (recorded in less than one-third of cases) and GCS scores (two-thirds of cases) from acute to rehabilitation contexts.

Bombardier et al. (6) investigated of the validity of a self-report TBI screening tool (Step 2 of the TBI-4 structured interview [27]) and compared self-report data with diagnosis by application of an algorithm based on CT, references to the GCS, LOC, PTA, and changes in mental state reported in medical records. The TBI-4 was found to overestimate both the incidence and severity of TBI by 80% and had only moderate sensitivity and poor specificity compared with TBI diagnosis made by application of the algorithm. It was concluded this method of screening would need to be significantly modified before it could be recommended for clinical use. One study included searching for references to cognitive impairment in medical records reviews (25) to identify persons with a co-occurring TBI admitted to a United States Veterans SCI unit. There was no difference in rates of cognitive impairment in the dual-diagnosis group compared with an SCI-only group.

In terms of timing of TBI ascertainment, few studies clearly identified the point in the patient pathway where TBI was first captured or commented on optimal timing of TBI assessment. Davidoff et al. (23) found that LOC was assessed in majority of patients in ED and rehabilitation settings (87% in ED, 67% in rehabilitation), but PTA was rarely assessed (22% in ED, 14% in rehabilitation). In another study, 40% of the dual-diagnosis group had TBI first identified in the ED, a third in acute care, and the rest while in inpatient rehabilitation (22). Five studies considered TBI diagnosis retrospectively during post-acute rehabilitation (5, 6, 11, 28, 29). Two studies noted the confounding influences of intoxication, medication, and physical complications on accuracy of acute TBI diagnosis (6, 26).

### Neuroradiology approaches to TBI screening (Table II)

Two studies evaluated imaging methods to identify TBI in persons with SCI (28, 29). Wei et al. (29) compared MRI data in the subacute period and subsequent diffusion-tensor imaging (DTI) derived fractional anisotropy (FA) data in white matter tracts between healthy control (*n* = 12), SCI-only (*n* = 15), and SCI with co-occurring TBI (*n* = 7) groups. Dual-diagnosis participants were found to have multiple regions of white matter (WM) with reduced FA, but no significant differences were found between SCI-only and control groups. Jang et al. (28) similarly conducted DTI scans on a tSCI group (*n* = 14) with cognitive impairment but with no lesions on MRI, matched to healthy controls (*n* = 30). The values of FA and fibre volume in the motor and cognitive tracts of the tSCI group were significantly lower than those of the control group (*p* < 0.05), suggesting axonal injury of these neural pathways.

### Summary of findings

Multiple TBI indicators together, rather than reliance on a single TBI marker such as the GCS, appear more reliable in identifying co-occurring TBI. The biggest issue identified by many studies was incomplete documentation of TBI indicators across episodes of care, likely contributing to underestimation of the incidence of co-occurring TBI. Motor vehicle accident and fall mechanisms were associated with higher likelihood of co-occurring severe TBI and inconsistent documentation of TBI indicators suggested higher rates of missed TBIs in the mild range of severity. Algorithmic approaches prioritizing specific variables for TBI diagnosis (8) may be challenging to apply in practice due to incomplete information in clinical records (5). A self-report TBI symptom questionnaire, when used alone, over-estimated presence and severity of TBI. No studies commented on optimal timing for capturing co-occurring TBI in a patient’s pathway, but some studies noted impacts of confounders on early accurate evaluation of TBI indicators.

### Impact of co-occurring TBI and tSCI on rehabilitation outcomes

*Length of stay, cost, and discharge destination (Table III)*. Length of stay in rehabilitation was a key outcome parameter, but findings were mixed. Five studies reported longer stays in rehabilitation among dual-diagnosis groups (6, 14, 30–32). No differences in length of stay between dual-diagnosis and SCI-only groups were noted in 4 studies (33–36). Rehabilitation length of stay did not differ based on TBI severity (36).

Daily costs of inpatient rehabilitation, costs, Functional Independence Measure (FIM) change, and level of nursing care were greater among a dual-diagnosis group compared with SCI only (30). Conversely, Macciocchi et al. (34) found that cost of rehabilitation did not significantly differ between groups. In a study by Garlanger et al. (33), those with moderate–severe dual diagnosis were significantly less likely to discharge to own home than the SCI-only group. Gordan et al. (37) found more speech pathology services were used by those with dual diagnosis and length of stay was associated with discharge cognitive function.

Gober et al. (32) assessed hospital length of stay among children (0–18 years) with dual diagnosis compared with an SCI-only group. Having a dual diagnosis was associated with longer average lengths of stay (6 vs 4 days) and increased mean total hospital charges (US$124,198 vs US$98,089) when compared with isolated SCI. Differences in costs associated with severity of the TBI in the dual-diagnosis group were not reported.

### Functional outcomes (Table III)

Most studies evaluating functional outcomes used FIM scores (*n* = 8), reporting differences in motor, cognitive, or total FIM domains on admission and discharge. Bombardier et al. (6) found no significant difference in any FIM scores in the dual-diagnosis group on discharge from rehabilitation compared with those without TBI. Nott et al. (35) compared TBI-only, SCI-only, and dual-diagnosis groups and similarly found the dual-diagnosis and SCI groups achieved comparable FIM cognitive scores throughout rehabilitation, and these scores were significantly higher than FIM cognitive scores achieved by the TBI-only group. On rehabilitation discharge, FIM motor and cognitive scores were comparable among the SCI and dual-diagnosis groups, despite a high proportion of severe TBI.

Other studies show significantly lower FIM outcomes at discharge in dual-diagnosis groups. Mollayeva et al. (38) showed lower FIM cognitive functional gain from admission to discharge among a dual-diagnosis group compared with a TBI-only group. Sikka et al. (22) and Macciocchi et al. (34) demonstrated lower motor FIM, cognitive FIM, and total FIM scores with any severity of co-occurring TBI at admission and discharge compared with tSCI-only groups. Macciocchi et al. (34) also reported smaller motor FIM change from rehabilitation among the dual-diagnosis group despite having statistically equivalent motor FIM scores on admission. TBI severity as defined by the GCS or intracranial lesions did not predict response to treatment in the dual-diagnosis group.

In a study by Garlanger et al. (33), those with moderate–severe co-occurring TBI had significantly lower cognitive FIM scores on admission and discharge and lower motor FIM efficiency scores compared with those with SCI only. The mild TBI group showed no significant differences in FIM scores on admission or discharge compared with the SCI-only group. Ramamurthy et al. (36) conversely did not find any differences in functional independence gained from rehabilitation based on severity of TBI at 5 years post-injury.

Macciocchi et al. (14) differentiated TBI-related functional outcomes based on level of tSCI (tetraplegia vs paraplegia) and severity of TBI. Those with paraplegia and co-occurring severe TBI had significantly worse motor and cognitive outcomes than those without TBI. In the tetraplegia group, moderate/severe TBI negatively affected functional cognition and neuropsychological test performance.

### Cognitive outcomes (Table III)

Cognitive outcomes were the focus of 5 studies (23, 30, 39–41). Two older studies used the Halstead Category Test (HCT; evaluates deficits in attention, concentration, visual problem-solving, abstract reasoning, ability to adapt) (23, 41). Davidoff et al. (23) reported a non-significant trend towards poorer performance and a greater number of errors on the HCT among the dual-diagnosis group compared with SCI alone. Richards et al. (41) described cognitive recovery among tSCI and co-occurring TBI of any severity, consistent with TBI recovery expectations. Bradbury et al. (30) used neuropsychological testing (specifically measures of attention and speed of processing) and found performances were lower in the dual-diagnosis group but did not analyse findings on the basis of TBI severity. Macciocchi et al. (42) compared cognitive performance between 26 and 76 days post-injury for SCI-only and SCI with mild TBI groups. Those with co-occurring mild TBI did not show significantly greater impairment on any neuropsychological test. Dowler et al. (40) used cluster analysis of neuropsychological test performances among 91 people with tSCI and identified 6 SCI groups with distinct cognitive profiles. At least 2 groups demonstrated cognitive deficits consistent with co-occurring TBI (deficits in attention and processing speed, cognitive flexibility deficits, and memory) but again TBI severity was not considered.

### Complications (Table III)

Three studies evaluated rates of complications (6, 31, 43), such as pressure ulcers, urinary tract infections, psychiatric diagnoses, and mortality risk, with mixed results. Bombardier et al. (6) found that comorbid TBIs were not associated with higher complications at 1 year after injury. In contrast, De Melo Nato et al. (31) and Mirzaeva et al. (43) both identified more complications and a higher mortality rate among dual-diagnosis vs SCI-only groups. Although co-occurring mild TBI was more common in these study samples, authors did not report findings based on TBI severity.

### Employment outcomes (Table III)

Three studies included longer term employment outcomes (35, 44, 45). Clark et al. (45) analysed veterans grouped as dual diagnosis, history of TBI/no SCI, and no history of TBI or SCI. TBI history was associated with poorer clinical outcomes at time of assessment regardless of co-occurring SCI and both dual-diagnosis and TBI/no SCI groups endorsed comparable levels of unemployment. Boyle et al. (44) evaluated levels of paid employment reported by people with dual diagnosis during the first 5 years post-rehabilitation discharge to explore contextual factors that supported employment. There were no statistically significant differences in the social, physical, or attitudinal supports/barriers experienced by the employed and unemployed subgroups. Nott et al. (35) recruited dual-diagnosis, TBI-only, SCI-only groups at an average 3.6 years post-rehabilitation. Similar levels of anxiety and depression were reported by participants in all groups; however, TBI participants reported higher stress levels. All groups also demonstrated similar levels of psychosocial functioning, community reintegration, relationship, and employment status.

### Summary of findings

Although most studies were cohort designs, with small to modest sample sizes, there were trends to longer length of stays and higher costs among dual-diagnosis groups. Moreover, Garlanger et al. (33) highlighted that those with moderate–severe dual diagnosis were notably less likely to discharge to own home compared with a tSCI-only group. Functional outcomes were typically assessed with the FIM. While some studies found comparable FIM scores between dual-diagnosis and tSCI-only groups, others reported significantly worse FIM outcomes, especially among moderate to severe dual-diagnosis groups. Cognitive FIM scores were often noted as lower for those with dual diagnosis, suggesting the presence of cognitive impairment, possibly linked to TBI. Neuropsychological testing revealed mixed results in terms of cognitive performance among individuals with dual diagnosis, but again there were trends to lower cognitive function among dual-diagnosis groups. While outcomes associated with TBI severity were not analysed or were inconsistent, trends to larger functional impacts with greater severity of TBI were noted in some studies.

## DISCUSSION

We reviewed approaches to screening for co-occurring TBI among persons with tSCI as well as outcomes among those with dual diagnosis, to better understand the impacts of co-occurring TBI.

### Screening for co-occurring TBI

The biggest challenges for capturing co-occurring TBI appear related to reliance on retrospective clinical records hampered by incomplete and inconsistent documentation of TBI indicators. Diagnostic difficulties are also underscored by problems untangling TBI symptoms and factors that are associated with the SCI, with symptoms not necessarily specific to TBI, such as cognitive impairments and fatigue, complicating diagnosis confidence (7, 12, 13). This is accentuated for mild TBI relative to moderate and severe TBI, where symptoms and signs are subtle, and less obvious and complicated by other factors such as substance intoxication at time of injury (10, 46). Studies included in the review suggested greater ambiguity and so the risk of missed TBI diagnosis was more likely for mild TBI and for non-transport related injury mechanisms. No studies considered the optimal timing for TBI screening, with screening approaches in acute care contexts especially complicated by overlap between impacts of SCI and the nonspecific nature of many TBI symptoms (15, 26). Self-report measures to screen for TBI symptoms may have limited utility if used in isolation. Generally, studies demonstrated higher rates of TBI across the severity spectrum when multiple TBI indicators were considered (11, 26).

### Impacts of co-occurring TBI on tSCI outcomes

Impacts of co-occurring TBI on tSCI rehabilitation outcomes, while mixed, tended to be larger with greater severity of TBI. Longer length of stays and higher costs among dual-diagnosis groups were suggested, especially for those with moderate–severe TBI. In terms of functional outcomes, groups with co-occurring moderate and severe TBI and tSCI tended to demonstrate lower admission and discharge FIM scores compared with tSCI-only groups. Typically, functional impacts as measured with the FIM were not found among people with co-occurring mild TBI. Outcomes relevant to the individual may not however, be easily captured by the FIM, with this potentially less sensitive to mild TBI impacts (47).

In terms of long-term outcomes (>12 months) differences between dual-diagnosis and tSCI-only groups were not typically evident (6, 36). Employment status and psychosocial functioning varied across studies, with some reporting similar levels across dual-diagnosis, TBI-only, and tSCI-only groups (44, 45). These findings highlight the complexity of relationships between co-occurring TBI and tSCI and the need for a consistent set of outcomes and measures to better capture and understand these relationships. Only 1 included study evaluated outcomes among children sustaining co-occurring TBI and tSCI.

### Limitations and directions for future research

Our search was not limited by geographical location, date, or study design. However, as many studies were conducted in the United States, the limited geographical diversity may affect the global generalizability of findings, as healthcare systems, cultural factors, and access to care can vary significantly. We did not limit our search by publication date; the included studies were published between 1987 and 2023, potentially introducing bias due to changes in diagnostic criteria, treatment approaches, and rehabilitation strategies over this extended period. Technological advancements and changes in medical practices may have influenced the outcomes observed.

Heterogeneity in study designs can make it challenging to bring findings together and draw robust conclusions. Many studies employed observational cohort designs, with small to modest sample sizes potentially contributing to mixed findings. As well, variability in screening methods and outcome measures across studies may contribute to inconsistencies in the identification of TBI cases and mixed rehabilitation outcomes, especially for mild TBI. This is likely to be accentuated by variability across studies and debates in the literature regarding defining especially mild TBI (48). Consistent with the scoping review methods we employed, we did not review quality of included studies and we did not provide any quantitative synthesis of findings.

Several potential next steps can be considered to improve TBI diagnosis and clinical outcomes, all underscored by the importance of more consistent diagnostic criteria and documentation of acute TBI indicators. First, following the TBI diagnostic literature (15), considering high-risk injury mechanisms can alert SCI clinicians to TBI risk. These include transport-related accidents, falls, and cervical-level SCIs, although Budisin et al. (24) remind us TBIs can also occur among persons with other injury mechanisms and SCI levels. The recently published criteria set for TBI, especially mild TBI, could improve diagnostic consistency in dual-diagnosis research (48)

Second, multiple TBI indicators (e.g., imaging modalities, GCS, PTA, mental state changes, cognitive signs), together with TBI symptoms captured by self-report screening tools, may be helpful to triangulate a TBI diagnosis. In our own work we are evaluating the use of a validated self-report TBI screening tool as a standard data point in SCI rehabilitation to flag the possibility of co-occurring TBI. This tool captures risk factors for TBI (e.g., high-risk mechanism, TBI signs, and symptoms). To address ambiguity associated with mild TBI indicators, we are augmenting this with clinical notes review, brief cognitive screening, and a brief oculomotor exam incorporated into a multivariable TBI screening process. Eye movements are helpful in identifying mild TBI in particular, (49) but have not yet been considered in the SCI literature. While many other symptoms of mild TBI such as issues with attention, memory, depression, sleep, headache, balance, and dizziness could be attributed to SCI, eye movements such as accommodation, convergence, and saccades appear generally unaffected by SCI. Given the ease of administration of the ocular examination, availability of standardized screening tools, and the ability to differentiate ocular symptoms that commonly occur following mild TBI but not tSCI, this could be a helpful adjunct to TBI screening.

Only one study in this review focused on a paediatric population (32). Expanding research efforts to focus on the paediatric population with co-occurring TBI and tSCI is indicated. This could involve investigating the unique challenges and opportunities in the rehabilitation of children and adolescents with dual diagnosis, including the impact on cognitive development, educational outcomes, and psychosocial well-being.

Finally, rehabilitation outcomes in most included studies were evaluated using measures of function not particularly sensitive to TBI impacts, especially mild TBI. Incorporating patient-centred measures and outcomes sensitive to all severities of TBI, but especially mild TBI, would help with understanding the perspectives and priorities of individuals with dual diagnosis. Thus, prospective longitudinal studies that use multivariable approaches for capturing TBI, tracking rehabilitation progress, and using outcome measures sensitive to TBI of all severities, may help to advance understandings.

### Conclusion

Accurate identification of co-occurring TBI in the clinical setting is highly dependent on consistent documentation of TBI indicators from acute to rehabilitation contexts. Multivariable TBI screening approaches may help to triangulate a TBI diagnosis, including attending to high-risk injury mechanisms, objective markers of TBI, and measures sensitive to mild TBI. Optimal timing for TBI assessment is largely unknown, with assessment of acute TBI symptoms complicated by the effects of the SCI. Accordingly, considering diagnosis of concomitant TBI in the sub-acute rehabilitation phase may be more effective, ensuring TBI is included in rehabilitation planning. Impacts of co-occurring TBI appear greater relative to SCI alone but few studies have analysed outcomes based on TBI severity or incorporated outcomes measures sensitive to impacts of mild TBI.
